# Is High Cholesterol Deleterious? An Alternative Point of View. Comment on Burén et al. A Ketogenic Low-Carbohydrate High-Fat Diet Increases LDL Cholesterol in Healthy, Young, Normal-Weight Women: A Randomized Controlled Feeding Trial. *Nutrients* 2021, *13*, 814

**DOI:** 10.3390/nu13062119

**Published:** 2021-06-21

**Authors:** Uffe Ravnskov

**Affiliations:** Independent Researcher, Magle Stora Kyrkogata 9, 22350 Lund, Sweden; uravnskov@gmail.com

In their study of the effect of an LCHF-diet on blood lipids, Burén et al. became worried because, after a few weeks, the participants’ LDL-cholesterol (LDL-C) increased significantly [1]. However, they need not worry. It is correct that many studies have found an association between LDL-C and cardiovascular disease, but association is not the same as causation. In a recent study, we have documented that the cholesterol hypothesis is unable to satisfy any of the Bradford Hill criteria for causality [2]. Accordingly, we have identified 38 studies where the authors have followed more than six million people of all ages for several years after having measured their LDL-C. In three of the studies, mortality was a little higher among those with the highest LDL-C, but mortality was highest among those with the lowest LDL-C. In the other 35 studies, those with the highest LDL-C lived just as long or, in most cases, longer than those with normal or low LDL-C [3,4]. 

The reason why high LDL-C is beneficial is most likely that LDL participates in our immune system by adhering to and inactivating all types of microorganisms and their toxic products, a little-known fact that has been documented in many ways by more than a dozen research groups [5,6].
